# Synthesis, Crystal Structure, DFT Studies and Evaluation of the Antioxidant Activity of 3,4-Dimethoxybenzenamine Schiff Bases

**DOI:** 10.3390/molecules19068414

**Published:** 2014-06-19

**Authors:** Ahmad Nazif Aziz, Muhammad Taha, Nor Hadiani Ismail, El Hassane Anouar, Sammer Yousuf, Waqas Jamil, Khalijah Awang, Norizan Ahmat, Khalid M. Khan, Syed Muhammad Kashif

**Affiliations:** 1 Atta-ur-Rahman Institute for Natural Product Discovery, Universiti Teknologi MARA, 42300 Puncak Alam Campus, Malaysia; E-Mails: nazif_umt@yahoo.com (A.N.A.); norhadiani@puncakalam.uitm.edu.my (N.H.I.), anouarelhassane@yahoo.fr (E.H.A.); 2 School of Fundamental Science, Universiti Malaysia Terengganu, 21030 Kuala Terengganu, Malaysia; 3 Faculty of Applied Sciences, Universiti Teknologi MARA, 40450 Shah Alam, Selangor, Malaysia; E-Mail: noriz118@salam.uitm.edu.my; 4 H.E.J. Research Institute of Chemistry, International Center for Chemical and Biological Sciences, University of Karachi, 75270 Karachi, Pakistan; E-Mails: dr.sammer.yousuf@gmail.com (S.Y.); khalid.khan@iccs.edu (K.M.K.); 5 Institute of Advance Research Studies in Chemical Sciences, University of Sindh Jamshoro, Hyderabad 76080, Pakistan; E-Mails: waqas143kh@yahoo.com (W.J.); hope_cancer@yahoo.com (S.M.K.); 6 Department of Chemistry, Faculty of Science, Universiti Malaya, 50603 Kuala Lumpur, Malaysia; E-Mail: khalijah@um.edu.my

**Keywords:** 3,4-dimethoxybenzenamine Schiff bases, crystal structure, DFT calculation, antioxidant activity

## Abstract

Schiff bases of 3,4-dimethoxybenzenamine **1**–**25** were synthesized and evaluated for their antioxidant activity. All the synthesized compounds were characterized by various spectroscopic techniques. In addition, the characterizations of compounds **13**, **15** and **16** were supported by crystal X-ray determinations and their geometrical parameters were compared with theoretical DFT calculations at the B3LYP level of theory. Furthermore, the X-ray crystal data of two non-crystalline compounds **8** and **18** were theoretically calculated and compared with the practical values of compounds **13**, **15**, **16** and found a good agreement. The compounds showed good DPPH scavenging activity ranging from 10.12 to 84.34 μM where compounds **1**–**4** and **6** showed stronger activity than the standard *n*-propyl gallate. For the superoxide anion radical assay, compounds **1**–**3** showed better activity than the standard.

## 1. Introduction

Schiff bases are a class of compounds with unique biological [1], analytical and industrial properties [2]. A number of Schiff bases have been reported to possess antiglycation [3,4,5,6], antioxidant [7,8,9,10], antileishmanial [11], antifungal [12], anticancer [13], anticonvulscent [14], analgesic [15], antituberclotic [16], and diuretic [17] activities. Heterocyclic Schiff bases with various activities e.g., antibacterial, antifungal and anticancer have also been reported [18,19,20]. The active pharmacophore (-N=CH-) of Schiff bases plays a major role in these significant biological activities. However, the attached neighbouring groups may also affect the activity [21]. Biological activities of Schiff bases metal complexes have also been reported [22,23]. The copper (II) complexes of Schiff bases showed antitumor activity and the lanthanide complexes showed significant antioxidant activity [24,25,26,27,28]. Antioxidants can prevent injury to vessel membranes aiding appropriate blood circulation and are useful for the prevention of cardiovascular diseases. They provide protection against cancer-causing radicals and DNA damages [29]. The action of antioxidants is credited to their ability to convert free radicals to stable molecules. Antioxidants therefore, guard cells from the oxidative damage which leads to aging and diseases [30,31,32]. The free radicals were also reported to play a role in the pathology of arteriosclerosis, malaria and rheumatoid arthritis [33,34]. 

As for polyphenolic compounds, the phenolic Schiff bases (ArOH) scavenge free radicals (R^•^) by their ability to donate hydrogen atoms from hydroxyl groups through one of the following mechanisms:

### (i) Proton Coupled-Electron Transfer (PC-ET) versus Hydrogen atom transfer (HAT)


ArOH + R^•^ → ArO^•^ + RH

In this mechanism, the electron and proton are transferred from the active phenolic group to the free radical in a single step. This type of reactions can be subdivided into two distinct subclasses, hydrogen atom transfer (HAT) and proton coupled electron transfer (PC-ET) [35,36,37,38]. In HAT, the proton and electron are transferred together, as a hydrogen atom, while in PC-ET mechanism, the proton and electron are transferred between different sets of orbitals [35]. 

### (ii) Electron Transfer-Proton Transfer (ET-PT)


ArOH + R^•^ →ArOH^+•^+ R^−^ → ArO^•^ + RH

The ET-PT mechanism consists of two steps. In the first step, an electron transfer (ET) from the phenolic compound to the free radical. In the second step, a heterolytic O-H bond dissociation of the radical cation (ArOH^+•^) leads to the formation of a phenoxyl radical (ArOH^•^).

### (iii) Sequential Proton Loss Electron Transfer (SPLET)


ArOH → ArO^−^ + H^+^ ArO^−^ + R^•^ → ArO^•^ + R^−^ R^−^ + H^+^ → RH

The above mechanism consists of three steps. In the first step, a heterolytic bond dissociation of a phenolic hydroxyl group leads to the formation of a phenoxyl anion and the release of a proton. In the second step, an electron transfer from the phenoxyl anion to the free radical leads to the formation of a phenoxyl radical and an anion (R^−^). In the end, the protonation of R^−^ leads to the formation of RH. This mechanism is strongly favored under alkaline conditions (e.g., high pH), which may help in the proton of the first step [39,40].

### (iv) Adduct formation (AF)


ArOH + R^•^ → [ArOH-R]^•^ → stable adducts

The AF mechanism is more specific and is observed between (a) carbon centered radicals and double bonds; or (b) hydroxyl radicals and aromatic rings. Numerous side reactions may occur that lead to stable adducts from [ArOH-R]^•^.

**Scheme 1:**
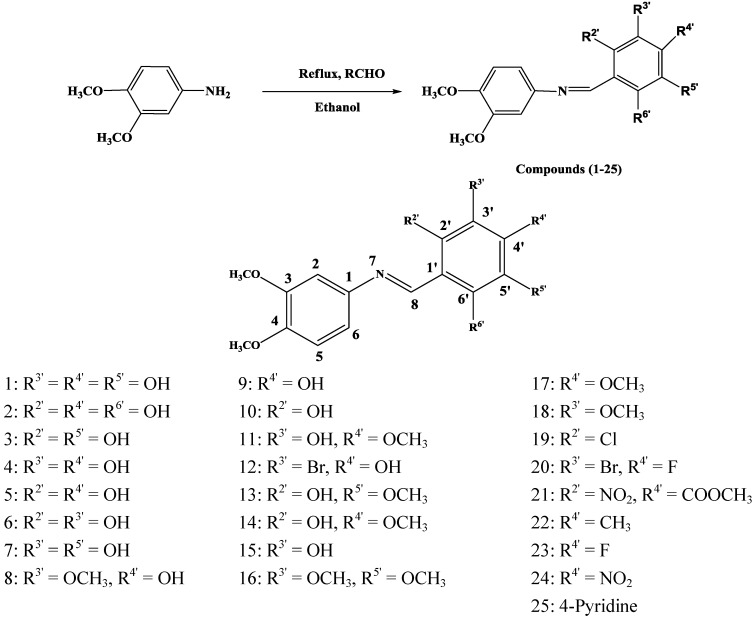
Synthesis of 3,4-dimethoxybenzenamine Schiff bases.

In continuation of our research on the synthesis of bioactive small molecules [41,42,43], we synthesized a series of 3,4-dimethoxybenzenamine Schiff bases (Scheme 1) and evaluated their antioxidant potential in the search of the potential antioxidant leads.

## 2. Results and Discussion

### 2.1. Chemistry

The 3,4-dimethoxybenzenamine Schiff bases were prepared by condensing 3,4-dimethoxy-benzenamine with several aromatic aldehydes by refluxing in ethanol for 3 to 4 h (Scheme 1). The crude products were further recrystallized from methanol and in most of the cases needle-like crystals were obtained (yields 81%–92%). The structural confirmation of the dimethoxybenzenamine Schiff bases was done by various spectroscopic techniques including ^1^H-NMR, IR and mass spectroscopy. All synthetic compounds were established as having *E* configuration [44,45,46]. The compounds **8**, **9**, **10**, **15**, **22**, **24** and **25** are known [47,48,49,50] but compound **13** has only a CAS registry number 1002275–90–2 with no reference. Compounds **1–7**, **11**, **12**, **14**, **16**, **18–21**, **23** are new.

### 2.2. Antioxidant Activities

#### 2.2.1. DPPH Scavenging Activity

The synthesized compounds **1–25** showed activity in the range of 10.12–84.34 μM (Table 1). Compound **1** (IC_50_ = 10.12 ± 0.54 μM) showed highest activity, three times more active than the standard (IC_50_ = 30.30 ± 0.2 μM). This is due to the *ortho*-trihydroxyl group which is known to show very good activity [51,52]. Compound **2** is a *meta*-trihydroxyl analogue but showed slightly less activity than compound **1**. This may be due to the *ortho*-trihydroxyl groups of compound **1** which is similar to the catecholic moeity known to exhibit good antioxidant activities [53,54,55,56]. 

**Table 1:** *In vitro* DPPH activity and % yield of compounds **1–25**.

N°	Yield (%)	IC_50_ (μM ± SEM ^a^)	N°	Yield (%)	IC_50_ (μM ± SEM ^a^)
**1**	84	10.12 ± 0.54	**14**	87	42.80 ± 2.80
**2**	82	15.6 ± 0.06	**15**	82	NA ^b^
**3**	78	19.2 ± 0.70	**16**	82	NA ^b^
**4**	84	28.14 ± 0.86	**17**	84	NA ^b^
**5**	85	30.45 ± 0.82	**18**	83	NA ^b^
**6**	86	28.10 ± 1.30	**19**	85	NA ^b^
**7**	81	33.02 ± 1.20	**20**	87	NA ^b^
**8**	83	34.14 ± 1.50	**21**	88	NA ^b^
**9**	92	40.01 ± 1.80	**22**	-	NA ^b^
**10**	88	NA ^b^	**23**	90	NA ^b^
**11**	90	50.01 ± 2.20	**24**	84	NA ^b^
**12**	87	38.16 ± 2.10	**25**	92	NA ^b^
**13**	90	NA ^b^	*n*-propyl gallate ^c^	-	30.30 ± 0.2

SEM ^a^ is the standard error of the mean, NA ^b^ = Not active, *n*-propyl gallate ^c^ was the standard drug for the DPPH assays.

Incidently, compounds **1** and **2** which have an additional hydroxyl group as compared to compounds **4** and **6**, showed stronger antioxidant activities than the latter two compounds. Among the five dihydroxyl analogues, compound **3**, **4** and **6** showed better activity than standard. The activity of compound **3** is due to the 2', 5' positions of the dihydroxyl groups, favorable for stabilization of the free radical. The catecholic moeity in compounds **4** and **6** is well known structural feature for good activity [53,54,55,56]. Other *meta*-dihydroxyl analogues **5** and **7** also showed very close activity as compared to the standard. Compound **8** showed good activity due to adjacent 3-methoxyl and 4-hydroxyl positions. However, its other analogue **11** with reversed arrangement showed moderate activity.

For the mono-hydroxyl series, compound **9** having hydroxyl at 4' position showed good activity while its other analogues **10** and **15** showed no activity, due to the lack of free radical stabilizing capability. The presence of a bromo substituent further increases the capability to stabilize radicals as illustrated by compound **12**. *meta*-arranged methoxyl and hydroxyl groups contributed to the good activity of compound **14**. The remaining compounds do not possess functional groups to help stabilize free radicals and are therefore inactive.

#### 2.2.2. Superoxide Scavenging Activity

Compounds **1**, **2** and **3** showed better activity than the standard drug *n*-propylgallate (Table 2). Compound 4 showed good activity. Compounds **5**, **6**, **7**, **8**, **9** and **12** showed moderate activities, while compound **14**, **20** and **21** showed weak activities. The good activity of compounds **1–3** may be due to more stabilizing potential of these compounds to stabilize free radicals generated during bioassay. DPPH scavenging activity and superoxide scavenging activity mainly depend on the hydroxyl position as well as number of hydroxyl groups present in the molecule [57,58].

**Table 2:** *In vitro* superoxide anion radical scavenging activity of compounds **1–25**.

Comp. No.	IC_50_ (μM ± SEM ^a^)	Comp. No	IC_50_ (μM ± SEM ^a^)
**1**	85.03 ± 1.20	**14**	260.3 ± 6.4
**2**	90.60 ± 1.50	**15**	NA ^b^
**3**	98.60 ± 1.70	**16**	NA ^b^
**4**	145 ± 2.1	**17**	NA ^b^
**5**	170.2 ± 3.2	**18**	NA ^b^
**6**	175.0 ± 3.5	**19**	NA ^b^
**7**	180.1 ± 3.8	**20**	315.1 ± 8.4
**8**	190.1 ± 3.9	**21**	320.1 ± 6.3
**9**	208.9 ± 5.4	**22**	NA ^b^
**10**	NA ^b^	**23**	NA ^b^
**11**	NA ^b^	**24**	NA ^b^
**12**	210.1 ± 4.4	**25**	NA ^b^
**13**	NA ^b^	*n*-propyl gallate ^c^	106.34 ± 1.6

SEM ^a^ is the standard error of the mean, NA ^b^ = Not active, *n*-propyl gallate ^c^ was the standard drug for the superoxide anionradical scavenging assays.

### 2.3. X-ray Crystallography Studies

#### 2.3.1. Compound **13**

**Figure 1:**
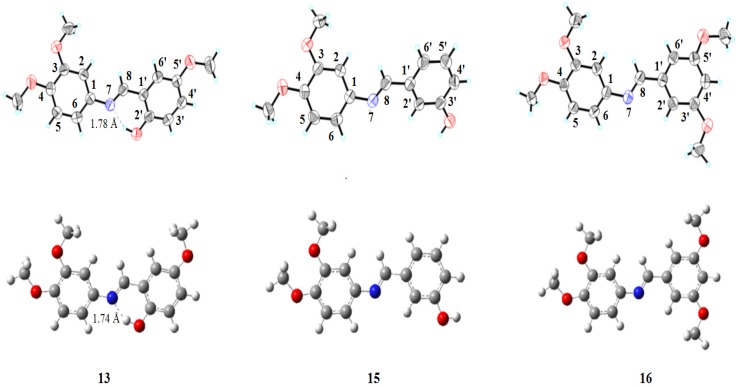
X-ray and optimized structures of compounds **13**, **15** and **16**.

**Figure 2:**
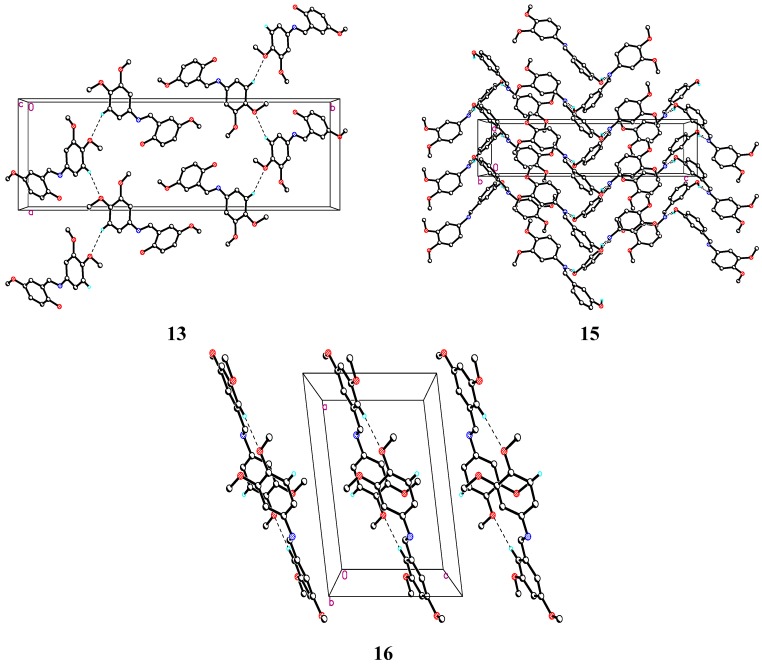
Crystal packing diagram for compounds **13**, **15** and **16**.

The structure of compound **13** is composed of a dimethoxybenzene moiety link with methoxyphenol moiety via azomethin bridge which adopts an *E* configuration (Figure 1). The dimethoxy-substituted planar benzene moiety (C1-C6) is oriented at a dihedral angle of 29.33(9)° with respect to the methoxy-substituted planar phenol moiety (C1'–C6') with standard deviation of 0.016(2)° for C2' atom from root mean square plane. In the crystal lattice (Figure 2), molecules are linked via C–H···O hydrogen bonding and form three dimensional consolidated network of mirror imaged sets running along the *b* axis where each set contains four molecule.

Crystallographic data of compound **13** (CCDC 980015), can be obtained from Cambridge Crystallographic Data Center without any cost. Crystal and experimental data of compound **13** are presented in Table 3 and the hydrogen bonding data in Table 4.

**Table 3:** The crystal X-ray and experimental data of compounds **13**, **15** and **16**.

	Compound 13	Compound 15	Compound 16
Empirical formula	C_16_H_17_NO_4_	C_15_H_15_NO_3_	C_17_H_19_NO_4_
Formula weight	287.31	257.28	301.33
Temperature	273(2)K	273(2)K	273(2)K
Wavelength	0.71073 Å	0.71073 Å	0.71073 Å
Crystal system	Orthorhombic	Orthorhombic	Monoclinic
Space group	Pna2(1)	P2(1)2(1)2(1)	P2(1)/c
a	9.7203(7) Å	5.3625(2)Å	11.1898(4) Å
b	30.576(2) Å	11.1755(5) Å	17.5567(6) Å
c	4.8328(3) Å	21.9532(10)Å	8.1013(3) Å
*α*	90°	90°	90°
*β*	90°	90°	98.0720(10)°
*γ*	90°	90°	90°
Volume	1436.36(17)A^3^	1315.63(10)A^3^	1575.78(10) A^3^
Z	4	4	4
Calculated density	1.329 mg/m^3^	1.299 mg/m^3^	1.270 mg/m^3^
Absorption coefficient	0.096 mm^−1^	0.091 mm^−1^	0.091 mm^−1^
F(000)	608	544	640
Crystal size	0.67 × 0.16 × 0.14 mm	0.77 × 0.49 × 0.45 mm	0.46 × 0.44 × 0.42 mm
θ range	1.33 to 25.50 °	1.86 to 25.50°	1.84 to 25.50
Reflections Collected	8206	7839	9213
Reflections Unique	2629	2426	2934
(*R*_int_)	0.0216	0.0165	0.0148
*R*_1_ with I > 2σ(I)	0.0347	0.0369	0.0352
*R*_2_ with I > 2σ(I)	0.0808	0.1066	0.0967
*R*_1_ for all data	0.0412	0.0386	0.0399
*R*_2_ for all data	0.0855	0.1086	0.1012
Goodness of fit	1.059	1.091	1.046
max/min *ρ* eA°^−3^	0.107 and −0.129	0.349 and −0.295	0.140 and −0.141
CCDC number	CCDC 980015	CCDC 980014	CCDC 980016

**Table 4:** Hydrogen bonding data for compound 13.

D	H	A	D-H	H...A	D...A	D-H…A
O1'	H1A'	N7	0.94(3)	1.78(3)	2.628(2)	149(2)
C5	H5A	O2 ^a^	0.93	2.53	3.319(2)	143

Symmetry codes: ^a^ 1/2+x,1/2-y,z.

#### 2.3.2. Compound **15**

Structurally, compound **15** is similar to compound **13** with the difference that the planar phenyl ring (C1'-C6') has only one hydroxyl group at the C3' position. The dihedral angle between the two planar benzene ring (C1'-C6' and C1-C6) was found to be 44.35(7)° with standard deviation of 0.009(2)° for C5 atom from root mean square plane.

The molecule does not possess any intramolecular interactions in the crystal lattice, molecules are packed in a series and form a consolidated network running along *c*-axis, joining through intermolecular O1---H1A'...N7, C6---H6'A...O2, C5---H5A...O1 hydrogen bonds. Crystallographic data of compound **15** (CCDC 980014), can be obtained from Cambridge Crystallographic Data Center without any cost. Crystal and experimental data of compound 15 presented in Table 3 and hydrogen bonding data in Table 5.

**Table 5:** Hydrogen bonding data for compound **15**.

D	H	A	D-H	H...A	D...A	D-H…A
O1'	H1A'	N7 ^a^	0.939(18)	1.891(18)	2.7841(16)	158.1(16)
C6'	H6'A	O2 ^b^	0.93	2.58	3.2572(17)	130
C5	H5A	O1 ^c^	0.93	2.57	3.1655(18)	123

Symmetry codes: ^a^ 1/2+x,1/2-y,-z ; ^b^ 1-x,1/2+y,1/2-z; ^c^ -3/2+x,1/2-y,-z.

#### 2.3.3. Compound **16**

Compound **16** is structurally similar to Compound **13** and Compound **15** with the only difference that all four substituents on the aromatic skeleton are methoxyl groups. In this molecule the two benzene rings and the azomethine group are practically coplanar and the molecule adopts an *E* configuration about the C8-N7 bond. The dihedral angle between the two planar benzene rings (C1'-C6' and C1-C6) was found to be 40.56(6)° with standard deviation of −0.019(1)° for C1 atom from root mean square plane. All the bond distances are within normal range comparable to those of similar compounds. No chemical intramolecular interaction was observed. However in the crystal structure, molecules were linked via C2-H2'A...O1 and C3-H3A...O1 intermolecular interactions to form R^2^_2_ (20) ring motive running along c-axis.

The crystallographic data of compound **16** (CCDC 980016), can be obtained from Cambridge Crystallographic Data Center. Crystal and experimental data of compound **16** presented in Table 3 and hydrogen bonding data in Table 6.

**Table 6:** Hydrogen bonding data for compound **16**.

D	H	A	D-H	H...A	D...A	D-H…A
C2	H2’A	O1 ^a.^	0.93	2.57	3.4945(16)	176
C3	H3A	O1 ^b^	0.93	2.52	3.4418(16)	170

Symmetry codes: ^a^ 1-x,-y,2-z; ^b^ x,1/2-y,-1/2+z.

### 2.4. DFT Calculations

**Figure 3:**
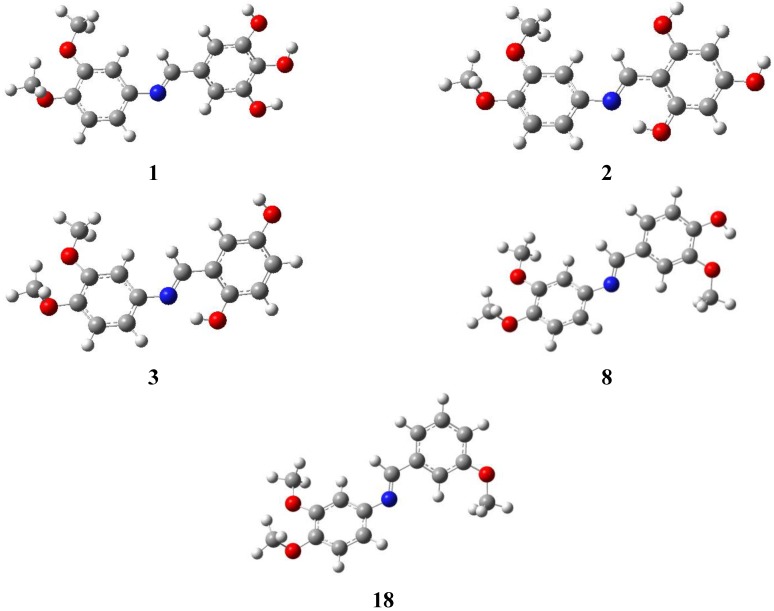
Optimized structures of compounds **1**, **2**, **3**, **8** and **18**.

Crystal structures of compounds **13**, **15** and **16** were compared to their optimized minima (Figure 3). The initial geometrical structures were obtained from the molden files of the X-ray solved structures. The optimization has been carried out at the B3LYP/6-311+G(d,p) level of theory by using Gaussian 09 package [7]. 

The minima of the optimized structures were confirmed by the absence of imaginary frequencies. The experimental and calculated bond lengths, bond and dihedral angles of the compounds are presented in Table 7. 

**Table 7:** The calculated and experimental values of the bond lengths, bond angles and torsion angles of compounds **13**, **15**, and **16**.

	13		15		16		Calculated				
	Cal	Exp	Cal	Exp	Cal	Exp	1	2	3	8	18
**Bond lengths (Å)**											
C1-N7	1.41	1.413	1.40	1.4243	1.41	1.4181	1.40	1.41	1.41	1.40	1.41
N7-C8	1.29	1.276	1.28	1.2673	1.28	1.2677	1.28	1.29	1.29	1.27	1.28
C8-C1'	1.45	1.452	1.47	1.4680	1.47	1.4650	1.47	1.44	1.45	1.46	1.47
C2'-O2'	1.34	1.358	-	-	-	-	-	1.37	1.35	-	-
C3'-O3'	1.37	-	1.37	1.3601	1.36	1.3673	1.36	-	-	1.37	1.36
C5'-O4'	-	-	-	-	-	-	1.37	1.36	-	1.36	-
C5'-O5'	1.37	1.382	-	-	1.37	1.3694	1.38	-	1.37	-	-
C6'-O6'	-	-	-	-	-	-	-	1.34	-	-	-
**Bond angles (°)**											
C2-C1-N7	125	123.31	126	122.15	126	122.79	123	123	123	123	123
C1-N7-C8	124	121.07	123	119.72	123	118.20	121	122	122	120	121
N7-C8-C1’	122	122.43	122	123.96	123	124.02	123	122	122	123	123
**Torsion angles (°)**											
C2-C1-N7-C8	0	−155.52	0	−43.09	0	34.70	37	32	34	36	30
C1-N7-C8-C1'	−180	−175.32	−180	174.26	−180	−179.25	−177	−177	−177	−177	−177
N7-C8-C1'-C2’	0	0	0	−1.1	0	−174.83	2	0	1	1	1

The experimental bond lengths and bond angles z-matrix coordinates are well reproduced theoretically. On the other hand, the dihedral angles are well reproduced, except for the dihedral angle between the 3,4-dimethoxybenzenyl and the azo group where a slight deviation was observed between the crystal structure compared to the optimized conformation. The hydrogen bonding between 2'-OH group and the azo group in Compound **13** is well reproduced theoretically, with a difference of 0.04 Å (Figure 1). In order to generalize the comparison between structural X-ray and calculated results, the optimized structures of compounds **1**, **2**, **3**, **8** and **18** were obtained at the same level of theory (Table 7 and Figure 3). The structural parameters (bond, angles, and torsion angles) for the optimized structures of **1**, **2**, **3**, **8** and **18** are very similar to the optimized and X-ray parameters of **13**, **15** and **16**.

## 3. Experimental

### 3.1. General Information

NMR experiments were performed in DMSO-*d_6_* on a Bruker Ultra Shield 500 MHz FT NMR (Wissembourg, Switzerland). CHN analysis was performed on a Carlo Erba Strumentazione-Mod-1106 (Milan, Italy). Electron impact mass spectra (EI-MS) were recorded on a Finnigan MAT-311A instrument (Bremen, Germany). Thin layer chromatography (TLC) was performed on pre-coated silica gel aluminum plates (Kieselgel 60, 254, E. Merck, Darmstadt, Germany). Chromatograms were visualized by UV at 254 and 365 nm.

### 3.2. DPPH (1,1-Diphenyl-2-picryl hydrazyl) Free Radical Scavenging Activity

The free radical scavenging activity was measured by the 1,1-diphenyl-2-picrylhydrazyl (DPPH) assay using literature protocols. The reaction mixture contained test sample (5 μL, 1 mM in DMSO) and DPPH (Sigma, 95 μL, 300 μM) in ethanol. The reaction mixture was taken into a 96-well microtiter plate and incubated at 37 °C for 30 min. The absorbance was measured at 515 nm using microtitre plate reader (Molecular Devices, Sunnyvale, CA, USA). Percent radical scavenging activity was determined in comparison with DMSO containing control (Table 1). IC_50_ values represent the concentration of compounds able to scavenge 50% of DPPH radicals. Propyl gallate was used as positive control. All chemicals used were of analytical grade (Sigma, Ronkonkoma, NY, USA).

### 3.3. In Vitro Assay for Superoxide Anion Radical Scavenging Activity

The superoxide producing system was set up by mixing phenazinemethosulfate (PMS), NADH, and oxygen (air), and the production of superoxide was estimated by the nitroblue tetrazolium method. Measurement of superoxide radical scavenging activity was carried out on the basis of the method described by the modified method used by Ferda. In aerobic reaction mixtures containing NADH, phenazine methosulphate and nitro blue tetrazolium, PMS is reduced by NADH and then gave rise to O_2_^−^, which in turn reduced NBT. On the basis of this PMS has frequently been used to mediate O_2_^−^.

The reaction mixture comprised 100 µM β-nicotinamide adenine dinucleotide reduced form (NADH, 40 µL), 80 µM of nitro blue tetrazolium (NBT, 40 µL), 8 µM phenazine methosulphate (PMS, 20 µL), 1 mM sample (10 µL), and 0.1 M phosphate buffer (pH 7.4, 90 µL). The reagents were prepared in buffer and sample in DMSO. The reaction was performed in 96-well microtitre plate at room temperature and absorbance was measured at 560 nm. The formation of superoxide was monitored by measuring the formation of water soluble blue formosan dye. A lower absorbance of reaction mixture indicated a higher scavenging activity of the sample. Percent radical scavenging activity (% RSA) by samples was determined in comparison with a control using the following equation:


%RSA = 100 − {(OD test compound/OD control) × 100

### 3.4. General Procedure for the Synthesis 3,4-Dimethoxybenzenamine Schiff Bases

The 3,4-dimethoxyanaline Schiff bases were synthesized by refluxing in ethanol (10 mL) for 3 h 3,4-dimethoxyanaline (2 mmol) and each pure aryl aldehyde (2 mmol). The progress of the reaction was monitored by TLC. After completion of reaction, the solvent was evaporated under vacuum to afford crude products which were further recrystallized from methanol to give needle-like pure products in good to excellent yields.

*(E)-5-(((3,4-Dimethoxyphenyl)imino)methyl)benzene-1,2,3-triol* (**1**). ^1^H-NMR: *δ* 11.05 (s, 2H, OH), 9.61 (s, 1H, OH), 8.60 (s, 1H, N=CH-Ar), 7.05 (s, 2H), 6.95 (d, 1H, *J* = 8.0 Hz), 6.91 (d, 1H, *J* = 2.0 Hz), 6.82 (dd, 1H, *J* = 8.0, *J* = 2.0, Hz), 3.83 (s, 3H, OCH_3_), 3.77 (s, 3H, OCH_3_); ^13^C-NMR: *δ* 160.1, 150.1, 148.1, 146.3, 146.2, 146.2, 138.1, 134.2, 119.4, 114.2, 109.2, 108.8, 108.8, 56.3, 56.3; Anal. Calcd for C_15_H_15_NO_5_, C = 62.28, H = 5.23, N = 4.84 Found C = 62.29, H = 5.22, N = 4.85 EI MS *m/z* (% rel. abund.): 289 (M^+^, 10), 258 (12), 138 (20), 137 (100).

*(E)-2-(((3,4-Dimethoxyphenyl)imino)methyl)benzene-1,3,5-triol* (**2**). ^1^H-NMR: *δ* 10.65 (s, 2H, OH), 9.23 (s, 1H, OH), 8.65 (s, 1H, N=CH-Ar), 7.15 (s, 2H), 6.94 (d, 1H, *J* = 8.0 Hz), 6.90 (d, 1H, *J* = 2.0 Hz), 6.81 (dd, 1H, *J* = 8.0, *J* = 2.0 Hz), 3.82 (s, 3H, OCH_3_), 3.79 (s, 3H, OCH_3_); ^13^C-NMR: *δ* 163.7, 163.7, 163.4, 160.1, 146.4, 150.2, 148.1, 119.4, 114.3, 109.2, 106.1, 96.2, 96.2, 56.1, 56.1; Anal. Calcd for C_15_H_15_NO_5_, C = 62.28, H = 5.23, N = 4.84, Found C = 62.28, H = 5.23, N = 4.84; EI MS *m/z* (% rel. abund.): 289 (M^+^, 13), 258 (11), 138 (17), 137 (100).

*(E)-2-(((3,4-Dimethoxyphenyl)imino)methyl)benzene-1,4-diol* (**3**). ^1^H-NMR: *δ* 12.49 (s, 1H, OH), 10.18 (s, 1H, OH), 9.19 (s, 1H, N=CH-Ar), 7.11 (d, 1H, *J* = 7.0 Hz), 7.04 (s, 1H), 7.00 (d, 1H, *J* = 7.0 Hz), 6.97 (d, 1H, *J*_3/2_ = 8.0 Hz), 6.86 (d, 1H, *J* = 8.0, Hz), 6.78 (s, 1H), 3.80 (s, 3H, OCH_3_), 3.78 (s, 3H, OCH_3_); ^13^C-NMR: *δ* 160.0, 153.6, 151.0, 150.1, 148.7, 146.3, 120.3, 119.4, 119.5, 118.4, 116.1, 114.2, 109.2, 56.1, 56.1; Anal. Calcd for C_15_H_15_NO_4_, C = 65.92, H = 5.53, N = 5.13, Found C = 65.91, H = 5.54, N = 5.12; EI MS *m/z* (% rel. abund.): 273 (M^+^, 60), 241 (8), 137 (100), 122 (20), 109 (30).

*(E)-4-(((3,4-Dimethoxyphenyl)imino)methyl)benzene-1,2-diol* (**4**). ^1^H-NMR: *δ* 12.20(s, 1H, OH), 10.30 (s, 1H, OH), 8.40 (s, 1H, N=CH-Ar), 7.10 (d, 1H, *J* = 7.0 Hz), 7.06 (s, 1H), 7.02 (d, 1H, *J* = 7.0 Hz), 6.88 (d, 1H, *J*_3/2_ = 8.0 Hz), 6.82 (d, 1H, *J* = 8.0, Hz), 6.74 (s, 1H), 3.83 (s, 3H, OCH_3_), 3.79 (s, 3H, OCH_3_); ^13^C-NMR: *δ* 149.4, 146.2, 117.1, 116.1, 131.1, 123.1, 160.2, 146.4, 109.1, 114.2, 119.1, 148.1, 150.0, 56.1, 56.1; Anal. Calcd for C_15_H_15_NO_4_, C = 65.92, H = 5.53, N = 5.13, Found C = 65.90, H = 5.55, N = 5.11; EI MS *m/z* (% rel. abund.): 273 (M^+^, 42), 241 (12), 137 (100), 122 (15), 109 (28).

*(E)-4-(((3,4-Dimethoxyphenyl)imino)methyl)benzene-1,3-diol* (**5**). ^1^H-NMR: *δ* 13.71 (s, 1H, OH), 10.18 (s, 1H, OH), 8.80 (s, 1H, N=CH-Ar), 7.40 (d, 1H, *J* = 8.0 Hz), 7.05 (d, 1H, *J* = 2.0 Hz), 6.99 (d, 1H, *J* = 7.0 Hz), 6.92 (dd, 1H, *J* = 8.0, *J* = 2.0, Hz), 6.40 (dd, 1H, *J* = 8.0, *J* = 2.0, Hz), 6.28 (d, 1H, *J* = 2.0 Hz),3.82 (s, 3H, OCH_3_), 3.78 (s, 3H, OCH_3_); ^13^C-NMR: *δ* 162.3, 162.1, 160.1, 150.2, 148.1, 146.4, 133.6, 119.4, 114.2, 113.0, 109.1, 108.5, 103.5, 56.1, 56.1; Anal. Calcd for C_15_H_15_NO_4_, C = 65.92, H = 5.53, N = 5.13 Found C = 65.93, H = 5.55, N = 5.12; EI MS *m/z* (% rel. abund.): 273 (M^+^, 70), 241 (17), 137 (100), 122 (22), 109 (38).

*(E)-3-(((3,4-Dimethoxyphenyl)imino)methyl)benzene-1,2-diol* (**6**). ^1^H-NMR: *δ* 13.42 (s, 1H, OH), 9.11 (s, 1H, OH), 8.93 (s, 1H, N=CH-Ar), 7.72 (d, 1H, *J* = 8.0 Hz), 7.15 (d, 1H, *J* = 8.0 Hz), 7.10 (s, 1H), 7.07 (d, 1H, *J* = 2.0 Hz), 6.76 (dd, 1H, *J* = 8.0, *J* = 2.0, Hz), 7.65 (t, 1H, *J* = 8.0 Hz), 3.81 (s, 3H, OCH_3_), 3.79 (s, 3H, OCH_3_); ^13^C-NMR: *δ* 160.1, 151.5, 150.1, 148.2, 146.4, 146.0, 124.6, 122.7, 119.8, 119.6, 119.5, 114.2, 109.1, 56.1, 56.1; Anal. Calcd for C_15_H_15_NO_4_, C = 65.92, H = 5.53, N = 5.13, Found C = 65.91, H = 5.54, N = 5.12; EI MS *m/z* (% rel. abund.): 273 (M^+^, 78), 241 (16), 137 (100), 122 (11).

*(E)-5-(((3,4-Dimethoxyphenyl)imino)methyl)benzene-1,3-diol* (**7**). ^1^H-NMR: *δ* 9.47 (s, 2H, 2×OH), 8.45 (s, 1H, N=CH-Ar), 7.01 (d, 2H, *J* = 8.0 Hz), 6.84 (d, 1H, *J* = 2.0 Hz), 6.65 (d, 1H, *J* = 8.0 Hz), 6.40 (d, 1H, *J* = 2.0 Hz), 6.06 (dd, 1H, *J* = 8.0, *J* = 2.0, Hz), 3.81 (s, 3H, OCH_3_), 3.78 (s, 3H, OCH_3_); ^13^C-NMR: *δ* 160.1, 160.1, 160.0, 150.1, 148.1, 146.4, 141.3, 119.5, 114.3, 109.1, 107.4, 107.4, 105.8, 56.1, 56.1; Anal. Calcd for C_15_H_15_NO_4_, C = 65.92, H = 5.53, N = 5.13, Found C = 65.92, H = 5.54, N = 5.11; EI MS *m/z* (% rel. abund.): 273 (M^+^, 50), 241 (11), 137 (100), 122 (18).

*(E)-4-(((3,4-Dimethoxyphenyl)imino)methyl)-2-methoxyphenol* (**8**). ^1^H-NMR: *δ* 9.65 (s, 1H, OH), 8.49 (s, 1H, N=CH-Ar), 7.51 (d, 1H, *J* = 2.0 Hz), 7.32 (dd, 1H, *J* = 8.0, *J* = 2.0, Hz), 6.96 (d, 1H, *J* = 8.0 Hz), 6.93 (d, 1H, *J* = 2.0 Hz), 6.89 (d, 1H, *J* = 8.0 Hz), 6.06 (dd, 1H, *J* = 8.0, *J* = 2.0 Hz), 3.85 (s, 3H, OCH_3_), 3.81 (s, 3H, OCH_3_), 3.76 (s, 3H, OCH_3_); ^13^C-NMR: *δ* 160.1, 151.1, 150.1, 149.1, 148.2, 146.3, 130.8, 122.8, 119.5, 117.1, 114.2, 112.0, 109.1, 56.1, 56.1, 55.9; Anal. Calcd for C_16_H_17_NO_4_, C = 66.89, H = 5.96, N = 4.88, Found C = 65.90, H = 5.95, N = 4.90; EI MS *m/z* (% rel. abund.): 287 (M^+^, 100), 255 (13), 137 (84), 122 (25).

*(E)-4-(((3,4-Dimethoxyphenyl)imino)methyl)phenol* (**9**). ^1^H-NMR: *δ* 10.04 (s, 1H, OH), 8.50 (s, 1H, N=CH-Ar), 7.77 (d, 2H, *J* = 8.0 Hz), 6.96 (d, 1H, *J* = 8.0 Hz), 6.92 (d, 1H, *J* = 2.0 Hz), 6.88 (d, 2H, *J* = 8.0 Hz), 6.81 (dd, 1H, *J* = 8.0, *J* = 2.0, Hz), 3.80 (s, 3H, OCH_3_), 3.76 (s, 3H, OCH_3_); ^13^C-NMR: *δ* 160.5, 160.0, 150.0, 148.1, 146.4, 130.4, 130.4, 129.2, 119.4, 116.0, 116.0, 114.2, 109.1, 56.1, 56.1; Anal. Calcd for C_15_H_15_NO_3_, C = 70.02, H = 5.88, N = 5.44, Found C = 70.01, H = 5.89, N = 5.43; EI MS *m/z* (% rel. abund.): 257 (M^+^, 100), 225 (11), 137 (68), 105 (20).

*(E)-2-(((3,4-Dimethoxyphenyl)imino)methyl)phenol* (**10**). ^1^H-NMR: *δ* 13.32 (s, 1H, OH), 8.93 (s, 1H, N=CH-Ar), 7.63 (dd, 1H, *J* = 8.0, *J* = 2.0 Hz), (dt, 1H, *J* = 8.0, *J* = 2.0 Hz), 6.93 (d, 1H, *J* = 2.0 Hz), 6.80-6.72 (m, 4H), 3.83 (s, 3H, OCH_3_), 3.78 (s, 3H, OCH_3_); ^13^C-NMR: *δ* 161.1, 160.0, 150.1, 148.1, 146.4, 132.2, 132.0, 121.3, 120.4, 119.4, 117.6, 114.2, 109.1, 56.1, 56.1; Anal. Calcd for C_15_H_15_NO_3_, C = 70.02, H = 5.88, N = 5.44, Found C = 70.03, H = 5.88, N = 5.43; EI MS *m/z* (% rel. abund.): 257 (M^+^, 100), 225 (15), 137 (80), 93 (30).

*(E)-5-(((3,4-Dimethoxyphenyl)imino)methyl)-2-methoxyphenol* (**11**). ^1^H-NMR: *δ* 9.28 (s, 1H, OH), 8.48 (s, 1H, N=CH-Ar), 7.42 (d, 1H, *J* = 2.0 Hz), 7.30 (dd, 1H, *J* = 8.0, *J* = 2.0, Hz), 7.04 (d, 1H, *J* = 8.0 Hz), 6.97 (d, 1H, *J* = 8.0 Hz), 6.93 (d, 1H, *J* = 2.0 Hz), 6.82 (dd, 1H, *J* = 8.0, *J* = 2.0, Hz), 3.84 (s, 3H, OCH_3_), 3.80 (s, 3H, OCH_3_), 3.77 (s, 3H, OCH_3_); ^13^C-NMR: *δ* 160.0, 152.1, 150.1, 148.2, 147.2, 146.4, 147.1, 131.0, 125.4, 119.4, 115.8, 114.2, 109.1, 56.1, 56.1, 55.8; Anal. Calcd for C_16_H_17_NO_4_, C = 66.89, H = 5.96, N = 4.88, Found C = 66.88, H = 5.95, N = 4.89; EI MS *m/z* (% rel. abund.): 287 (M^+^, 100), 255 (18), 137 (69), 122 (20).

*(E)-2-Bromo-4-(((3,4-dimethoxyphenyl)imino)methyl)phenol* (**12**). ^1^H-NMR): *δ* 12.68 (s, 1H, OH), 8.94 (s, 1H, N=CH-Ar), 8.04 (d, 1H, *J* = 2.0 Hz), 7.76 (dd, 1H, *J* = 8.0, *J* = 2.0, Hz), 7.07 (d, 1H, *J* = 8.0 Hz), 6.98 (d, 1H, *J* = 8.0 Hz), 6.95 (d, 1H, *J* = 2.0 Hz), 6.84 (dd, 1H, *J* = 8.0, *J* = 2.0 Hz), 3.80 (s, 3H, OCH_3_), 3.77 (s, 3H, OCH_3_); ^13^C-NMR (DMSO-*d_6_*): *δ* 160.1, 158.6, 150.1, 148.2, 146.4, 130.2, 129.5, 128.4, 119.5, 118.1, 114.3, 113.8, 109.2, 56.1, 56.1; Anal. Calcd for C_15_H_14_BrNO_3_, C = 53.59, H = 4.20, N = 4.17, Found C = 53.60, H = 4.21, N = 4.18; EI MS *m/z* (% rel. abund.): 337 (M+2 , 61), 335 (M^+^ , 64), 255 (30), 137 (100).

*(E)-2-(((3,4-Dimethoxyphenyl)imino)methyl)-4-methoxyphenol* (**13**). ^1^H-NMR): *δ* 10.91 (s, 1H, OH), 8.52 (s, 1H, N=CH-Ar), 7.22 (d, 1H, *J* = 2.0 Hz), 7.12 (d, 1H, *J* = 2.0 Hz), 7.03–6.97 (m, 3H), 6.90 (d, 1H, *J* = 8.0 Hz), 3.84 (s, 3H, OCH_3_), 3.80 (s, 3H, OCH_3_), 3.76 (s, 3H, OCH_3_); ^13^C-NMR: *δ* 160.1, 153.2, 153.1, 150.1, 148.2, 146.4, 119.5, 118.2, 118.0, 117.1, 114.2, 113.4, 109.2, 56.1, 56.1, 55.8; Anal. Calcd for C_16_H_17_NO_4_, C = 66.89, H = 5.96, N = 4.88, Found C = 66.91, H = 5.95, N = 4.91; EI MS *m/z* (% rel. abund.): 287 (M^+^, 100), 255 (19), 137 (80), 122 (25).

*(E)-2-(((3,4-Dimethoxyphenyl)imino)methyl)-5-methoxyphenol* (**14**). ^1^H-NMR): *δ* 13.84 (s, 1H, OH), 8.88 (s, 1H, N=CH-Ar), 7.51 (d, 1H, *J* = 7.5 Hz), 7.09 (d, 1H, *J* = 2.0 Hz), 7.01 (d, 1H, *J* = 8.0 Hz), 6.96 (dd, 1H, *J* = 8.0, *J* = 2.0 Hz), 6.57 (dd, 1H, *J* = 7.5, *J* = 2.0 Hz), 6.48 (dd, 1H, *J* = 2.0 Hz), 3.83 (s, 3H, OCH_3_), 3.81(s, 3H, OCH_3_), 3.78 (s, 3H, OCH_3_); ^13^C-NMR: *δ* 164.2, 162.0, 160.1, 1501, 148.2, 146.4, 133.2, 119.5, 114.2, 112.6, 109.1, 107.1, 103.3, 56.1, 56.1, 55.7; Anal. Calcd for C_16_H_17_NO_4_, C = 66.89, H = 5.96, N = 4.88, Found C = 66.90, H = 5.95, N = 4.92; EI MS *m/z* (% rel. abund.): 287 (M^+^, 100), 255 (30), 137 (85), 122 (19).

*(E)-3-(((3,4-Dimethoxyphenyl)imino)methyl)phenol* (**15**). ^1^H-NMR): *δ* 9.64 (s, 1H, OH), 8.57 (s, 1H, N=CH-Ar), 7.35 (d, 1H, *J* = 2.0 Hz), 7.31-7.30 (m, 2H), 7.31–7.30 (m, 2H), 6.98–6.97 (m, 1H), 6.92 (dd, 1H, *J* = 8.0, *J* = 2.0 Hz), 6.86 (dd, 1H, *J* = 7.5, *J* = 2.0 Hz), 3.82 (s, 3H, OCH_3_), 3.78 (s, 3H, OCH_3_); ^13^C-NMR (DMSO-*d_6_*): *δ* 160.1, 158.4, 150.1, 148.2, 146.4, 138.6, 130.1, 121.6, 119.5, 118.1, 114.8, 114.3, 109.2, 56.1, 56.1; Anal. Calcd for C_15_H_15_NO_3_, C = 70.02, H = 5.88, N = 5.44, Found C = 70.03, H = 5.91, N = 5.42; EI MS *m/z* (% rel. abund.): 257 (M^+^, 100), 225 (21), 137 (60), 93 (20).

*(E)-N-(3,5-Dimethoxybenzylidene)-3,4-dimethoxyaniline* (**16**). ^1^H-NMR): *δ* 8.60 (s, 1H, N=CH-Ar), 7.10 (d, 2H, *J* = 2.0 Hz), 7.00 (d, 1H, *J* = 2.0 Hz), 6.98 (d, 1H, *J* = 8.0 Hz), 6.96 (dd, 1H, *J* = 8.0, *J* = 2.0 Hz), 6.86 (t, 1H, *J* = 5.5 Hz), 3.82 (s, 9H, 3×OCH_3_), 3.78 (s, 3H, OCH_3_); ^13^C-NMR: *δ* 161.5, 161.5, 160.1, 150.1, 148.2, 146.4, 140.5, 119.5, 114.3, 109.2, 103.4, 103.4, 102.6, 56.1, 56.1, 55.8, 55.8; Anal. Calcd for C_17_H_19_NO_4_, C = 67.76, H = 6.36, N = 4.65, Found C = 67.77, H = 6.35, N = 4.66; EI MS *m/z* (% rel. abund.): 301 (M^+^, 40), 269 (45), 149 (21), 137 (100).

*(E)-3,4-Dimethoxy-N-(4-methoxybenzylidene)aniline* (**17**). ^1^H-NMR): *δ* 8.79 (s, 1H, N=CH-Ar), 8.10 (d, 2H, *J* = 8.5 Hz), 8.06 (d, 2H, *J* = 8.5 Hz),7.07 (d, 1H, *J* = 2.0 Hz), 7.02 (d, 1H, *J* = 8.0 Hz), 6.97 (dd, 1H, *J* = 8.0, *J* = 2.0 Hz), 3.89 (s, 3H, OCH_3_), 3.83 (s, 3H, OCH_3_), 3.80 (s, 3H, OCH_3_); ^13^C-NMR (DMSO-*d_6_*): *δ* 162.7, 160.1, 150.1, 148.3, 146.4, 130.1, 130.1, 128.5, 119.5, 114.3, 114.3, 114.2, 109.2, 56.1, 56.1; Anal. Calcd for C_16_H_17_NO_3_, C = 70.83, H = 6.32, N = 5.16, Found C = 70.84, H = 6.33, N = 5.15; EI MS *m/z* (% rel. abund.): 271 (M^+^, 26), 151 (11), 138 (17), 137 (100).

*(E)-3,4-Dimethoxy-N-(3-methoxybenzylidene)aniline* (**18**). ^1^H-NMR): *δ* 8.46 (s, 1H, N=CH-Ar), 7.46 (d, 1H, *J* = 8.0 Hz), 7.43 (d, 1H, *J* = 7.5 Hz),7.31-7.24 (m, 2H), 7.04 (d, 1H, *J* = 8.0 Hz), 6.98 (dd, 1H, *J* = 8.0, *J* = 2.0 Hz), 6.72 (d, 1H, *J* = 8.0 Hz), 3.84 (s, 3H, OCH_3_), 3.81 (s, 3H, OCH_3_), 3.80 (s, 3H, OCH_3_); ^13^C-NMR: *δ* 160.5, 160.1, 150.1, 148.3, 146.4, 138.1, 129.7, 121.4, 119.5, 116.4, 114.3, 111.3, 109.2, 56.1, 56.1; Anal. Calcd for C_16_H_17_NO_3_, C = 70.83, H = 6.32, N = 5.16, Found C = 70.82, H = 6.32, N = 5.15; EI MS *m/z* (% rel. abund.): 271 (M^+^, 100), 239 (20), 137 (60), 105 (24).

*(E)-N-(2-Chlorobenzylidene)-3,4-dimethoxyaniline* (**19**). ^1^H-NMR): *δ* 8.90 (s, 1H, N=CH-Ar), 8.16 (dd, 1H, *J* = 6.0, *J* = 2.0 Hz), 7.60 (dd, 1H, *J* = 6.0, *J* = 2.0 Hz), 7.56 (ddd, 1H, *J* = 6.0, *J* = 2.0, *J* = 2.0 Hz), 7.46 (t, 1H, *J* = 8.0 Hz), 7.02 (d, 1H, *J* = 7.5 Hz), 7.00 (s, 1H), 6.93 (dd, 1H, *J* = 6.0, *J* = 2.0 Hz), 3.84 (s, 3H, OCH_3_), 3.80 (s, 3H, OCH_3_); ^13^C-NMR: *δ* 157.1, 150.0, 148.2, 146.4, 133.8, 133.2, 132.2, 130.0, 127.1, 126.8, 119.5, 114.3, 109.2, 56.1, 56.1; Anal. Calcd for C_15_H_14_ClNO_2_, C = 65.34, H = 5.12, N = 5.08, Found C = 65.33, H = 5.13, N = 5.09; EI MS *m/z* (% rel. abund.): 277 (M+2, 31), 275 (M^+^, 100), 244 (15), 239 (17), 137 (40).

*(E)-N-(4-Bromo-3-fluorobenzylidene)-3,4-dimethoxyaniline* (**20**). ^1^H-NMR): *δ* 8.92 (s, 1H, N=CH-Ar), 7.90 (d, 1H, *J* = 8.0 Hz), 7.60 (dd, 1H, *J* = 7.0, *J* = 4.0 Hz), 7.20 (d, 1H, *J* = 8.0 Hz), 7.04 (d, 1H, *J* = 2.0 Hz), 6.98 (s, 1H), 6.93 (dd, 1H, *J* = 7.0, *J* = 2.0 Hz), 3.95 (s, 3H, OCH_3_), 3.83 (s, 3H, OCH_3_), 3.80 (s, 3H, OCH_3_); ^13^C-NMR: *δ* 165.4, 160.1, 148.2, 150.1, 146.4, 134.2, 133.2, 124.0, 119.5, 116.1, 114.2, 112.3, 109.2, 56.1, 56.1; Anal. C_15_H_13_BrFNO_2_, C = 53.27, H = 3.87, N = 4.14, Found C = 53.29, H = 3.89, N = 4.15; EI MS *m/z* (% rel. abund.): 339 (M+2, 46), 337 (M^+^, 49), 257 (25), 137 (100).

*(E)-Methyl 4-(((3,4-dimethoxyphenyl)imino)methyl)-3-nitrobenzoate* (**21**). ^1^H-NMR): *δ* 8.92 (s, 1H, N=CH-Ar), 8.70 (d, 1H, *J* = 2.0 Hz), 8.25 (dd, 1H, *J* = 7.0, *J* = 2.0 Hz), 8.21 (d, 1H, *J* = 8.0 Hz), 7.06 (d, 1H, *J* = 2.0 Hz), 7.01 (s, 1H), 6.96 (dd, 1H, *J* = 7.0, *J* = 2.0 Hz), 3.95 (s, 3H, OCH_3_), 3.83 (s, 3H, OCH_3_), 3.80 (s, 3H, OCH_3_); ^13^C-NMR: *δ* 165.8, 160.2, 150.1, 148.3, 147.6, 146.4, 136.1, 132.6, 131.6, 130.1, 123.4, 119.5, 114.2, 109.2, 56.1, 56.1, 52.1; Anal. Calcd for C_17_H_16_N_2_O_6_, C = 59.30, H = 4.68, N = 8.14, Found C = 59.31, H = 4.69, N = 8.15; EI MS *m/z* (% rel. abund.): 344 (M^+^, 100), 297 (22), 284 (30), 137 (100).

*(E)-3,4-Dimethoxy-N-(4-methylbenzylidene)aniline* (**22**). ^1^H-NMR): *δ* 8.61 (s, 1H, N=CH-Ar), 7.82 (d, 2H, *J* = 8.0 Hz), 7.33 (d, 2H, *J* = 8.5 Hz), 6.98 (d, 1H, *J* = 2.0 Hz), 6.97 (d, 1H, *J* = 8.0 Hz), 6.87 (dd, 1H, *J* = 8.0, *J* = 2.0 Hz), 3.81 (s, 3H, OCH_3_), 3.78 (s, 3H, OCH_3_), 2.38 (s, 3H, CH_3_); ^13^C-NMR: *δ* 160.1, 150.1, 148.2, 146.4, 140.5, 133.2, 129.2, 129.2, 129.0, 129.0, 119.6, 114.3, 109.2, 56.1, 56.1, 21.2; Anal. Calcd for C_16_H_17_NO_2_, C = 75.27, H = 6.71, N = 5.49, Found C = 75.29, H = 6.72, N = 5.50; EI MS *m/z* (% rel. abund.): 255 (M^+^, 100), 223 (18), 137 (80), 103 (28).

*(E)-3,4-Dimethoxy-N-(pyridin-4-ylmethylene)aniline* (**23**). ^1^H-NMR): *δ* 9.02 (s, 1H, N=CH-Ar), 8.61 (d, 2H, *J* = 8.0 Hz), 7.91 (d, 2H, *J* = 8.5 Hz), 7.03 (d, 1H, *J* = 2.0 Hz), 6.99 (d, 1H, *J* = 8.0 Hz), 6.91 (dd, 1H, *J* = 8.0, *J* = 2.0 Hz), 3.84 (s, 3H, OCH_3_), 3.79 (s, 3H, OCH_3_); ^13^C-NMR: *δ* 160.1, 150.2, 149.2, 148.3, 149.2, 144.2, 142.1, 120.3, 120.3, 119.5, 114.2, 109.3, 56.1, 56.1; Anal. Calcd for C_14_H_14_N_2_O_2_, C = 69.41, H = 5.82, N = 11.56, Found C = 69.40, H = 5.83, N = 11.57; EI MS *m/z* (% rel. abund.): 242 (M^+^, 100), 210 (20), 137 (50), 91 (50).

*(E)-N-(4-Fluorobenzylidene)-3,4-dimethoxyaniline* (**24**). ^1^H-NMR): *δ* 8.67 (s, 1H, N=CH-Ar), 8.00 (dd, 2H, *J* = 8.5, *J* = 4.0 Hz), 7.91 (t, 2H, *J* = 8.5 Hz), 7.00 (d, 1H, *J* = 2.0 Hz), 6.98 (d, 1H, *J* = 8.0 Hz), 6.94 (dd, 1H, *J* = 8.0, *J* = 2.0 Hz), 3.82 (s, 3H, OCH_3_), 3.78 (s, 3H, OCH_3_); ^13^C-NMR: *δ* 165.1, 160.1, 150.2, 148.3, 146.3, 132.1, 130.7, 130.7, 119.4, 115.5, 115.5, 114.3, 109.2, 56.1, 56.1; Anal. Calcd for C_15_H_14_FNO_2_, C = 69.49, H = 5.44, N = 5.40, Found C = 69.48, H = 5.43, N = 5.42; EI MS *m/z* (% rel. abund.): 259 (M^+^, 100), 227 (11), 137 (100), 95 (20).

*(E)-3,4-Dimethoxy-N-(4-nitrobenzylidene)aniline* (**25**). ^1^H-NMR): *δ* 8.88 (s, 1H, N=CH-Ar), 8.37 (d, 2H, *J* = 8.0 Hz), 8.18 (d, 2H, *J* = 8.5 Hz), 7.11 (d, 1H, *J* = 2.0 Hz), 7.03 (d, 1H, *J* = 8.0 Hz), 7.01 (dd, 1H, *J* = 8.0, *J* = 2.0 Hz), 3.84 (s, 3H, OCH_3_), 3.80 (s, 3H, OCH_3_), 2.38 (s, 3H, CH_3_); ^13^C-NMR: *δ* 160.1, 150.3, 150.1, 148.2, 146.4, 142.4, 127.6, 127.6, 124.1, 124.1, 119.5, 114.2, 109.2 Anal. Calcd for C_15_H_14_N_2_O_4_, C = 62.93, H = 4.93, N = 9.79, Found C = 62.94, H = 4.92, N = 9.80; EI MS *m/z* (% rel. abund.): 286 (M^+^, 100), 254 (17), 239 (20), 137 (100), 122 (16).

### 3.5. Theoretical Calculations

The optimization of the synthesized Schiff bases were performed at the B3LYP/6-311++G(d,p) level of theory [59]. The minima were confirmed by vibrational frequency analysis (*i.e.*, no imaginary frequency were found). All theoretical calculations were carried out using Gaussian09 package [60].

## 4. Conclusions

In conclusion, compounds having hydroxyl groups at suitable places as well as number of hydroxyl groups play a key role in the antioxidant activity. Three crystal structures along with its theoretical calculations are also reported with experimental value well correlated.
